# Exploring optical coherence tomography parameters in eyes with myopic tilted disc

**DOI:** 10.1186/s40662-024-00411-3

**Published:** 2024-11-02

**Authors:** Yu Qiao Zhang, Xiu Juan Zhang, Ru Yue Shen, Yuzhou Zhang, Fang Yao Tang, Simon K. H. Szeto, Danny Siu-Chun Ng, Ka Wai Kam, Alvin L. Young, Li Jia Chen, Chi Pui Pang, Clement C. Tham, Jason C. Yam, Poemen P. Chan

**Affiliations:** 1 Department of Ophthalmology and Visual Sciences, The Chinese University of Hong Kong, Hong Kong Eye Hospital, 147K Argyle Street, Kowloon, Hong Kong SAR, China; 2 Department of Ophthalmology, School of Clinical Medicine, LKS Faculty of Medicine, The University of Hong Kong, Hong Kong SAR, China; 3 Hong Kong Eye Hospital, Hong Kong SAR, China; 4 Department of Ophthalmology, Hong Kong Children’s Hospital, Hong Kong SAR, China; 5 Department of Ophthalmology and Visual Sciences, Prince of Wales Hospital, Hong Kong SAR, China; 6 Lam Kin Chung. Jet King-Shing Ho Glaucoma Treatment and Research Centre, Department of Ophthalmology and Visual Sciences, The Chinese University of Hong Kong, Hong Kong SAR, China; 7 Hong Kong Hub of Pediatric Excellence, The Chinese University of Hong Kong, Hong Kong SAR, China

**Keywords:** Myopic tilted disc, Myopia, Optical coherence tomography

## Abstract

**Background:**

To investigate the impact of optic disc torsion (ODT), horizontal disc tilt (HDT) angle, and ovality index (OI) on different retinal nerve fiber layer (RNFL) and ganglion cell-inner plexiform layer (GCIPL) segments in healthy myopic eyes.

**Methods:**

ODT and OI were measured from fundus photographs. HDT angle, peripapillary RNFL, and macular GCIPL were measured by swept-source optical coherence tomography (SS-OCT). The association between optic disc morphology and the RNFL/GCIPL thickness were evaluated, with age and axial length (AL) adjusted.

**Results:**

Among 530 healthy myopic eyes of 284 participants (mean age: 41.7 years, mean spherical equivalent: − 7.70 D, and mean AL: 26.6 mm), 335 eyes (63.2%) had temporal disc torsion (temporal group) and 195 eyes (36.8%) had nasal disc torsion (nasal group). For the nasal group, a larger OI was associated with thinner superior-to-superonasal GCIPL (β = − 7.465 to − 6.972, both *P* = 0.024) and temporal RNFL sectors (β = − 49.596 to − 27.748, *P* ≤ 0.014). For the temporal group, a larger OI was associated with thinner superior-to-nasal (β = − 50.255 to − 22.093, *P* ≤ 0.006) and thicker temporal RNFL sectors (β = 29.015 to 56.890, *P* ≤ 0.003). Additionally, a larger HDT angle was associated with thinner superior-to-nasal RNFL sectors (β = − 0.559 to − 0.242, *P* ≤ 0.036) and thinner superior-to-superotemporal GCIPL sectors (β = − 0.084 to − 0.069, *P* ≤ 0.037).

**Conclusions:**

The optic disc tortional direction was associated with the measurement of different RNFL and GCIPL sectors independent of the AL and age. These should be considered when constructing a myopic normative database.

**Supplementary Information:**

The online version contains supplementary material available at 10.1186/s40662-024-00411-3.

## Background

Myopia is one of the commonest global public health burdens with a prevalence of 80%–90% among young Asian adults [1] and a higher risk of developing blinding complications (e.g., glaucoma) [2–5]. Myopic tilted disc and axial elongation are common in myopia, jeopardize visual field (VF) and optical coherence tomography (OCT) measurements [6–10]. This often causes over-diagnosing or misdiagnosing of glaucoma [8, 11]. Myopic tilted disc could be measured by optic disc torsion (ODT), OCT horizontal disc tilt (OCT-HDT) angle, and the ovality index (OI) [12, 13]. Myopic ODT was associated with thicker temporal peripapillary retinal nerve fiber layer (RNFL) without affecting the macular ganglion cell-inner plexiform layer (GCIPL) thickness significantly [14–17]. Kim et al. showed that the position of the deepest point of the eyeball (DPE)—the most protruding posterior end of the globe at the deepest interface between the Bruch’s membrane and choroid—correlated with myopic tilted disc and ODT direction [12]. A more inferiorly located DPE was associated with an increased temporal ODT [18]. These scleral deformities also alter the adjacent RNFL/GCIPL distribution pattern, render identification of localized RNFL/GCIPL abnormalities difficult and make detection of pre-perimetric or early glaucoma in myopic eyes (often with reduced RNFL/GCIPL thickness [8] and unreliable VF results) challenging [19, 20]. The correlation between these changes and the direction of myopic ODT is not yet fully elucidated. Evaluating these changes in detail would enhance our understanding of different myopic optic disc anatomical variations, facilitating the construction of a more accurate normative database and diagnosis of myopic glaucoma.

In this study, the swept-source OCT (SS-OCT) and fundus photographs were utilized to quantify myopic tilted disc of healthy myopic eyes using ODT, OCT-HDT angle, and OI. The impact of these parameters on the RNFL/GCIPL measurements were analyzed.

## Methods

This was a cross-sectional study. The tenets of the Declaration of Helsinki were followed throughout. This study was approved by the Joint Chinese University of Hong Kong-New Territories East Cluster Clinical Research Ethics Committee (CREC Ref. No.: 2021.733). All participants joined the Hong Kong High Myopia Cohort from January 2017 to December 2021. They underwent best-corrected visual acuity (BCVA) measurement, intraocular pressure (IOP) measurement, slit-lamp examination, dark-room gonioscopy, and dilated fundi examinations. Axial length (AL) was obtained by optical low-coherence reflectometry (Lenstar, Haag-Streit AG, Koeniz, Switzerland). VF was performed with static automated white-on-white threshold perimetry (24–2 program, SITA-standard, Humphery Instruments, Dublin, California, USA). Only eyes with ≥ 2 reliable VF tests were included (fixation loss, false-positive and false-negative error of < 33%). Detailed medical history was also documented.

The inclusion criteria were: > 18 year-old with myopia, spherical equivalent (SE) of ≤ − 3.00 D, astigmatism within ± 4.00 D, BCVA of ≥ 20/25; optic nerve head without glaucomatous change, and normal VF results (Glaucoma Hemifield Test within normal limits, with the mean deviation and the pattern standard deviation beyond the probabilities of 5%). Eyes with other pathological changes (e.g., maculopathy), glaucoma, other optic neuropathy, history of ocular surgery/laser procedures, and participants who could not complete all the examinations, were excluded. Eligibility was determined by two glaucoma specialists (PPC and XJZ).

**Fig. 1:**
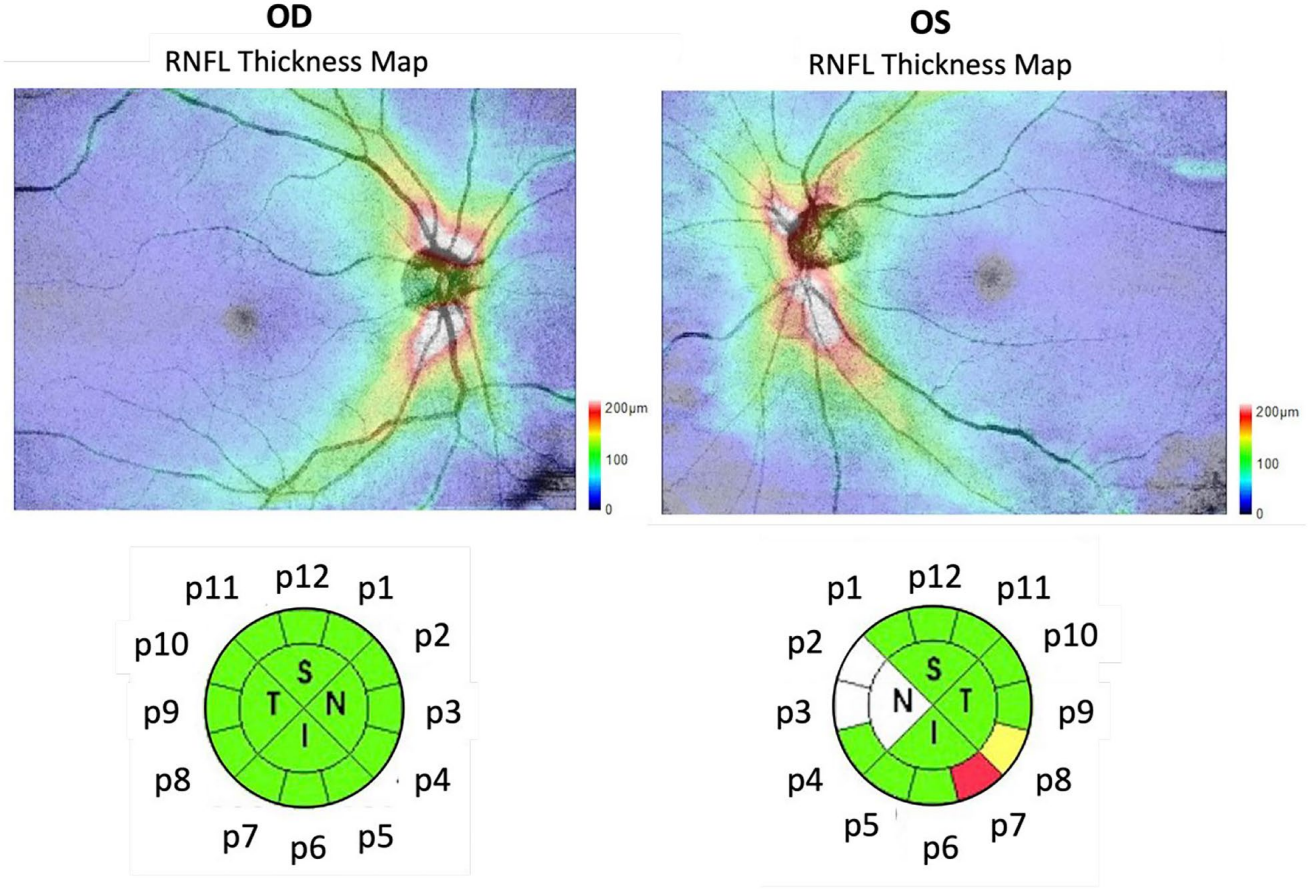
Segmentation of the peripapillary retinal nerve fiber layer (RNFL). The RNFL was segmented into p1–p12 in clockwise and anti-clockwise directions for the right and left eye, respectively

OCT images were taken by SS-OCT (DRI OCT Triton, Topcon, Japan), utilizing a 1,050 nm wavelength with a 100,000 A-scans/second scan speed. The 3D wide scan protocol covers a 12 × 9 mm area, which includes 256 B-scans, each comprising 512 A-scans (512 × 256 pixels). The images were then segmented by the built-in software for quantitative evaluation and generated an optic disc color photograph. A 3.4-mm diameter scan circle was automatically placed around the optic nerve head, RNFL was measured, and the thickness values of different sectors was provided. As both eyes were included in the study, the 12 o’ clocks of RNFL thickness map were labeled as p1 to p12 (Fig. 1). The macular layer was analyzed by a 6.0-mm circle centered on the fovea. The built-in software provided the macular GCIPL thickness and GCIPL plus macular RNFL thickness. Images with signal strength < 6, motion artifacts, poor centration, or poor focus were excluded. If no reliable image was obtained after three repeated OCT scans, the participant was excluded from the study.

**Fig. 2:**
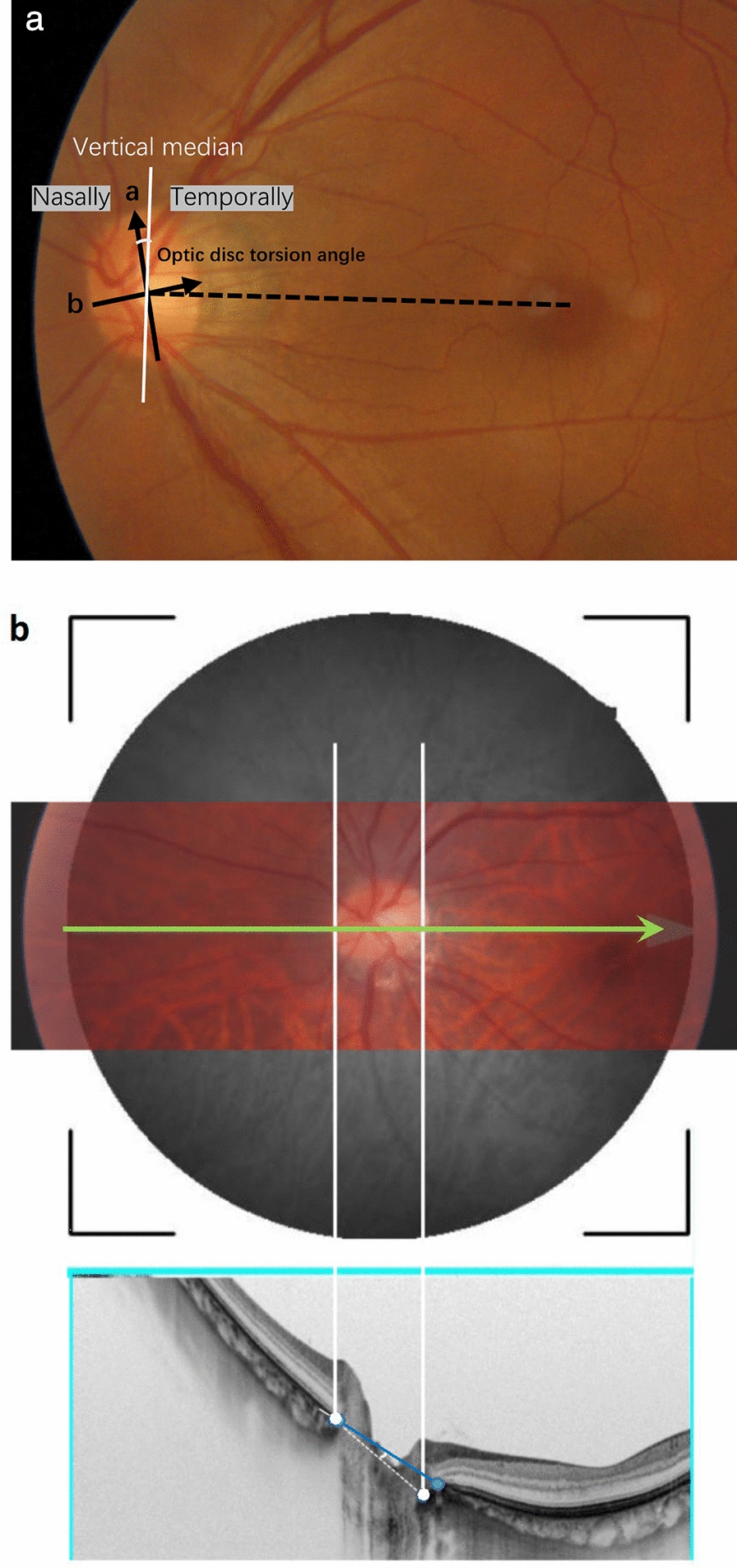
Measuring the parameters of myopic tilted disc: the ovality index, optic disc torsion, and optical coherence tomography (OCT) horizontal tilt angle. **a** The ovality index was determined by the ratio (a:b) of the longest diameter (arrow a) and the shortest diameter (arrow b) of the optic disc identified on the fundus photograph. The optic disc torsion angle was defined as the angle between the axis along the longest diameter of the optic disc (arrow a) and the vertical median (white line) that was perpendicular to the Disc-Fovea line (black dot line) connecting the center of the optic disc and the fovea. **b** The triple overlayed image consisted of an optic disc color photograph, the en-face swept-source OCT (SS-OCT) image, and the horizontal cross-sectional scan of the optic disc. According to the green line that crosses the optic disc, the clinical boundary of the optic disc was identified, with two white lines drawn across the clinical boundary and extended down to mark the corresponding optic disc border in the cross-sectional OCT scan. The white dots indicate the clinical boundary of the optic disc, and the two blue dots indicate the inner edges of the Bruch’s membrane. The OCT tilt angle was the angle between the connecting line of Bruch’s membrane border (blue line) and connecting line of the disc boundary (white dot line)

Two examiners (YQZ and RYS) independently measured the OI, ODT angle, and OCT-HDT angle using ImageJ (v.1.41, National Institutes of Health, USA), with methods described previously (Fig. 2) [21–23]; the average values were used for analysis. OI was the ratio of the optic disc’s longest and shortest diameters, reflecting the extent of optic disc tilting. The ODT angle was measured between the optic disc’s long axis and the line perpendicular to the Disc-Fovea line (Fig. 2a). The OCT-HDT angle was measured from a triple overlayed image (Fig. 2b) as the angle informed by the line connecting the optic disc borders and the line connecting the inner edges of the Bruch’s membrane opening (BMO) in the corresponding horizontal cross-sectional OCT scan**.** The Bruch’s membrane opening minimum rim width (BMO-MRW) was measured as previously described [24].

**Fig. 3:**
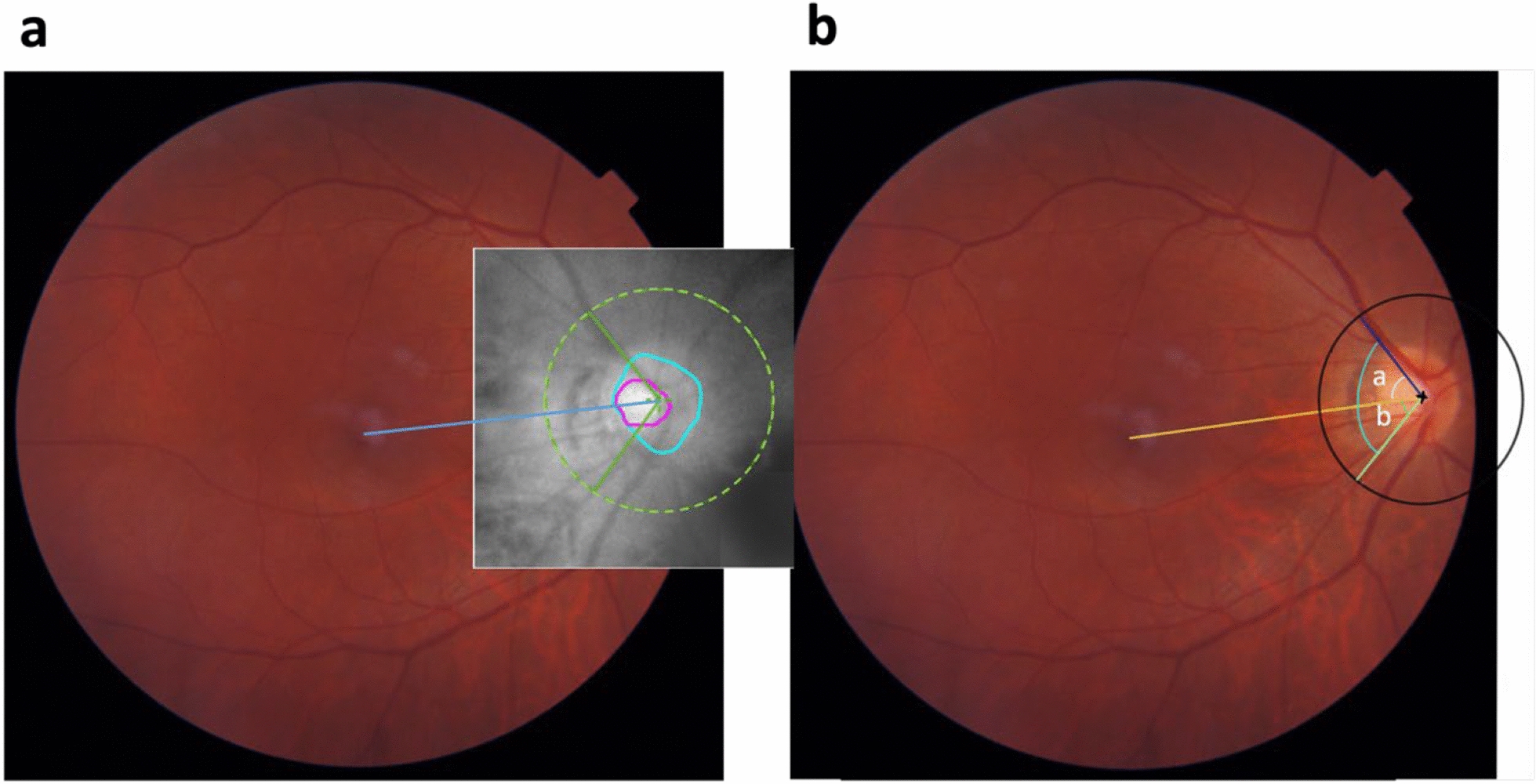
Measuring the superior and inferior retinal nerve fiber layer (RNFL) peak angle. Two en-face optical coherence tomography (OCT) images lining the superior and inferior peak of RNFL thickness were precisely overlapped with the corresponding color optic disc photograph (set to 50% transparency), guided by the retinal vessels’ location. **a** The triple overlayed image of the fundus photograph and two en-face swept-source-OCT images lining the superior RNFL peak and inferior RNFL peak (green lines), respectively. The blue line represents the Disc-Foveal line (connecting the fovea and center of the optic disc). **b** The measurement of the RNFL peak angle. The superior RNFL peak angle (angle a) was the angle between the Disc-Foveal line (yellow line) and the line that marked the location of the superior RNFL peak (blue line). Likewise, the inferior RNFL peak angle (angle b) was the angle between the Disc-Foveal line and the line that marked the inferior RNFL peak (green line)

The superior and inferior RNFL peaks’ locations were shown on the temporal-superior-nasal-inferior-temporal curve and the en-face optic disc OCT image (Fig. 3). The latter was overlapped with the corresponding optic disc photo as described. The RNFL peak angle was measured between the line of the RNFL peak (marked in the OCT image) and the Disc-Fovea line. The superior-inferior RNFL peak asymmetry was calculated by the superior RNFL peak thickness minus the inferior RNFL peak thickness.

Statistical analyses were performed using SPSS (v.26.0.02, IBM). Continuous, normally distributed variables were presented as mean ± SD. Categorical variables were presented as percentages. Participant-level variables were compared using the independent t-test. To account for inter-eye correlation of the same participant, eye-level variables were compared using generalized estimation equation (GEE). Age and AL were adjusted when applying GEE to compare the RNFL and GCIPL thickness between the two groups, and when determining the correlation between the myopic tilted disc parameters (OI, ODT angle, and OCT-HDT angle) and RNLF/GCIPL thickness. Interobserver reproducibility was calculated using intraclass correlation coefficient (ICC) with a two-way mixed effect model. The ICCs were categorized as poor (< 0.5), moderate (0.5 to 0.75), good (0.75 to 0.9), and excellent (> 0.9) [25]. A *P* value of less than 0.05 indicates statistical significance.

## Results

The study included 530 eyes (of 284 participants). Table 1 shows the baseline characteristics. 335 eyes (63.2%) had temporal ODT, and 195 eyes (36.8%) had nasal ODT.

**Table 1:** Demographic table of healthy myopic participants (530 eyes of 284 participants)

Parameter	Mean ± SD (range)
Age (years) (range)	41.7 ± 5.8 (25.0 to 72.0)
Axial length (mm) (range)	26.6 ± 1.4 (20.2 to 31.6)
Spherical equivalent (diopters) (range)	− 7.70 ± 3.11 (− 24.50 to − 3.50)
Vertical cup disc ratio	0.54 ± 0.18
OCT horizontal disc tilt angle (°)	14.63 ± 10.92
Optic disc torsion (ODT) angle (°)	21.31 ± 20.40
Ovality index	1.28 ± 0.15
Average GCIPL thickness (μm)	67.32 ± 4.97
Average RNFL thickness (μm)	100.46 ± 13.14

	Number of eyes (%)
ODT direction: temporal vs. nasal (%)	335 (63.2): 195 (36.8)

*OCT* = optical coherence tomography; *GCIPL* = macular ganglion cell-inner plexiform layer; *RNFL* = peripapillary retinal nerve fiber layer

Eyes were divided into temporal and nasal groups according to their ODT direction and were compared (Table 2) [26]. According to a previous study, temporal torsion was defined as temporal rotation of the optic disc’s longest axis from the vertical meridian, and nasal torsion was defined as nasal rotation of the optic disc’s longest axis from the vertical meridian. The temporal group was younger (41.1 ± 5.8 vs. 42.5 ± 5.6 years, *P* < 0.01), had longer AL (26.78 ± 1.24 vs. 26.28 ± 1.56 mm, *P* < 0.01), were more myopic (− 8.18 ± 2.71 vs. − 6.94 ± 3.55 D, *P* < 0.01), had larger OCT-HDT angle (16.07 ± 10.50° vs. 12.53 ± 11.23°, *P* < 0.01), ODT angle (23.53 ± 20.38° vs. 17.84 ± 20.00°, *P* < 0.01), and OI (1.30 ± 0.16 vs. 1.25 ± 0.13, *P* < 0.01). After adjusting for age and AL, the temporal group had thinner inferior (61.56 ± 5.58 vs. 63.48 ± 5.05 μm, *P* = 0.01) and inferonasal (67.38 ± 6.24 vs. 69.30 ± 5.30 μm, *P* = 0.03) GCIPL. They also had thinner superior (115.42 ± 22.64 vs. 123.87 ± 20.74 μm, *P* < 0.01), thinner nasal (58.89 ± 16.32 vs. 63.10 ± 18.30 μm, *P* = 0.02), and thicker temporal RNFL (101.97 ± 20.28 vs. 94.31 ± 18.79 μm, *P* < 0.01).

**Table 2:** Comparison of eyes with temporal disc torsion (temporal group) and nasal disc torsion (nasal group)

Parameter	Temporal group (n = 335)	Nasal group (n = 195)	*P* value
Age (years)	41.1 ± 5.8	42.5 ± 5.6	< 0.01*
Axial length (mm)	26.78 ± 1.24	26.28 ± 1.56	**< 0.01**^**†**^
Spherical equivalent (D)	− 8.18 ± 2.71	− 6.94 ± 3.55	**< 0.01**^**†**^
Rim area (mm^2^)	1.26 ± 0.48	1.84 ± 8.65	0.35^†^
Disc area (mm^2^)	1.90 ± 0.75	2.72 ± 11.63	0.30^†^
Vertical cup-disc ratio	0.47 ± 0.24	0.50 ± 0.24	0.15^†^
OCT horizontal tilt angle (°)	16.07 ± 10.50	12.53 ± 11.23	**< 0.01**^**†**^
Absolute value of optic disc torsion angle (°)	23.53 ± 20.38	17.84 ± 20.00	**< 0.01**^**†**^
Ovality index	1.30 ± 0.16	1.25 ± 0.13	**< 0.01**^**†**^
Bruch’s membrane opening-minimum rim width (μm)	269.79 ± 98.45	269.31 ± 88.38	0.96^†^
Macular GCIPL (μm)			
Superonasal	70.30 ± 5.68	71.51 ± 5.60	0.36^‡^
Inferonasal	67.38 ± 6.24	69.30 ± 5.30	**0.03**^‡^
Inferior	61.56 ± 5.58	63.48 ± 5.05	**0.01**^‡^
Inferotemporal	66.97 ± 6.86	68.72 ± 5.64	0.06^‡^
Superotemporal	67.34 ± 5.52	68.33 ± 5.30	0.37^‡^
Superior	66.97 ± 4.77	67.75 ± 5.08	0.56^‡^
Average	66.75 ± 5.04	68.18 ± 4.77	0.09^‡^
Peripapillary RNFL (μm)			
Superior	115.42 ± 22.64	123.87 ± 20.74	**< 0.01**^‡^
Nasal	58.89 ± 16.32	63.10 ± 18.30	**0.02**^‡^
Inferior	123.84 ± 23.57	125.76 ± 22.51	0.63^‡^
Temporal	101.97 ± 20.28	94.31 ± 18.79	**< 0.01**^‡^
Average	99.64 ± 13.56	101.70 ± 12.48	0.28^‡^

*OCT* = optical coherence tomography; *GCIPL* = ganglion cell inner plexiform layer; *RNFL* = retinal nerve fiber layer

^*^Comparison using independent t-test

^†^Comparison using generalized estimation equation (GEE)

^‡^Comparison using GEE adjusted by age and axial length

*P* values in bold indicate statistical significance

The associations between the myopic titled disc parameters and different GCIPL/RNFL sectors were evaluated (Table 3). The analyses were performed after adjusting for age, AL, and the two-neighbor myopic tilted disc parameters (e.g., additionally adjusted for OCT-HDT and ODT angle when analyzing the association between OI and RNFL thickness). Temporal and nasal ODT were presented as positive and negative values, respectively. A more temporally directed ODT correlated with thicker p9–10 (temporal) and thinner p12–1 RNFL (superior). A larger OCT-HDT angle correlated with thinner p12–2 (superior-to-superonasal) and p4–6 (inferior-to-inferonasal) RNFL. A larger OI correlated with thinner p12–5 (superior-to-nasal) and thicker p7–10 (temporal-to-inferior) RNFL.

**Table 3:** Relationship between optic disc morphology and OCT parameters (adjusted by axial length)

	Optic disc torsion angle (°)^a^		OCT horizontal tilt angle (°)^b^		Ovality index^c^	
Variables	Coefficient (95% CI)	*P* value	Coefficient (95% CI)	*P* value	Coefficient (95% CI)	*P* value
Macular GCIPL (μm)						
Superonasal	− 0.30 × 10^−2^ (− 0.02, 0.01)	0.72	− 0.05 (− 0.10, 0.08)	0.09	− 4.55 (− 8.29, − 0.82)	**0.02**
Inferonasal	− 0.01 (− 0.03, 0.01)	0.38	− 0.05 (− 0.11, 0.02)	0.20	− 2.61 (− 6.62, 1.40)	0.20
Inferior	− 0.90 × 10^−2^ (− 0.03, 0.01)	0.30	− 0.02 (− 0.07, 0.04)	0.58	− 3.63 (− 7.18, − 0.08)	**0.05**
Inferotemporal	− 0.40 × 10^–2^ (− 0.02, 0.02)	0.70	− 0.03 (− 0.10, 0.04)	0.48	− 3.49 (− 8.25, 1.26)	0.15
Superotemporal	− 0.30 × 10^−2^ (− 0.02, 0.02)	0.76	− 0.07 (− 0.13, − 0.01)	**0.02**	− 1.77 (− 5.82, 2.28)	0.39
Superior	− 0.10 × 10^−2^ (− 0.02, 0.02)	0.94	− 0.05 (− 0.09, 0.01)	0.05	− 3.34 (− 6.78, 0.10)	0.06
Peripapillary RNFL (μm)						
p1	− 0.11 (− 0.20, − 0.01)	**0.03**	− 0.40 (− 0.68, 0.13)	**< 0.01**	− 48.31 (− 67.53, − 29.09)	**< 0.01**
p2	− 0.06 (− 0.13, 0.01)	0.11	− 0.33 (− 0.54, − 0.11)	**< 0.01**	− 34.91 (− 49.28, − 20.55)	**< 0.01**
p3	− 0.01 (− 0.06, 0.04)	0.69	− 0.14 (− 0.30, 0.02)	0.08	− 24.10 (− 36.54, − 11.67)	**< 0.01**
p4	− 0.70 × 10^−2^ (− 0.07, 0.05)	0.82	− 0.19 (− 0.36, − 0.02)	**0.03**	− 33.98 (− 44.86, − 23.10)	**< 0.01**
p5	0.03 (− 0.06, 0.13)	0.52	− 0.39 (− 0.69, − 0.09)	**0.01**	− 36.16 (− 54.48, − 17.84)	**< 0.01**
p6	0.07 (− 0.04, 0.17)	0.21	− 0.36 (− 0.68, − 0.03)	**0.03**	− 14.23 (− 36.19, 7.74)	0.20
p7	0.04 (− 0.06, 0.14)	0.44	− 0.04 (− 0.41, 0.33)	0.85	24.58 (1.59, 47.55)	**0.04**
p8	− 0.01 (− 0.09, 0.07)	0.80	− 0.11 (− 0.39, 0.17)	0.44	37.74 (18.86, 56.62)	**< 0.01**
p9	0.06 (0.01, 0.12)	**0.03**	− 0.14 (− 0.32, 0.05)	0.15	32.03 (19.20, 44.86)	**< 0.01**
p10	0.14 (0.06, 0.23)	**< 0.01**	− 0.07 (− 0.36, 0.23)	0.64	49.86 (28.94, 70.78)	**< 0.01**
p11	− 0.03 (− 0.11, 0.06)	0.51	− 0.15 (− 0.44, 0.14)	0.32	4.41 (− 14.51, 23.34)	0.65
p12	− 0.12 (− 0.22, − 0.03)	**0.01**	− 0.43 (− 0.72, − 0.13)	**< 0.01**	− 19.00 (− 37.85, − 0.14)	**0.05**

*OCT* = optical coherence tomography; *GCIPL* = ganglion cell inner-plexiform layer; *RNFL* = retinal nerve fiber layer; *CI* = confidence interval

The p1–p12 represent the 12 segmentations of RNFL in clockwise and anti-clockwise directions for the right and left eye, respectively

In optic disc torsion angle, a positive value indicates optic disc torsion temporally, a negative value indicates optic disc torsion nasally. The generalized estimation equation was used, and it was adjusted for age, axial length, and other myopic tilted disc parameters (i.e., additionally adjusted for ^a^OCT horizontal tilt angle and ovality index, ^b^optic disc torsion angle and ovality index, and ^c^optic disc torsion angle and OCT horizontal tilt angle)

*P* values in bold indicate statistical significance

The temporal and nasal groups were evaluated separately (Table 4). For the temporal group, a larger ODT angle correlated with thinner p7–8 RNFL (β = − 0.18; CI: − 0.35 to − 0.010; *P* = 0.04 and β = − 0.18; CI: − 0.32 to − 0.04; *P* = 0.01, respectively) but was not associated with GCIPL thickness. A larger OCT-HDT angle correlated with thinner superior-to-superotemporal GCIPL (β = − 0.08; CI: − 0.16 to − 0.5 × 10^−2^ and β = − 0.07; CI: − 0.13 to − 0.01; *P* ≤ 0.04) and superior-to-nasal (p12–5) RNFL (all *P* ≤ 0.04); the association with the thinner p9 RNFL was marginal (*P* = 0.05). A larger OI correlated with thinner superior-to-nasal (p12–5) and thicker temporal RNFL (p8–10) (all *P* < 0.01). For the nasal group, a larger OI correlated with a thinner superior-to-superonasal GCIPL (β = − 6.97; CI: − 13.04 to − 0.91 and β = − 7.47; CI: − 13.95 to − 0.98; both *P* = 0.02), and temporal (p2–5) RNFL (all *P* ≤ 0.01). Neither the ODT angle nor OCT-HDT angle were associated with the GCIPL/RNFL thickness. The subgroup analyses based on myopia severity (< − 6.00 D and ≥ − 6.00 D) were performed for the temporal and nasal groups separately (Supplementary Tables 3 and 4). The correlation patterns of either subgroup were similar to the pre-stratification patterns. However, for the temporal group with severe myopia (SE < − 6.00 D), the OCT-HDT angle did not correlate with the GCIPL thickness and had fewer correlations with the RNFL sectors (a larger OCT-HDT angle correlated with thinner p1, p5, and p9 RNFL). Similarly, the general lack of correlation between ODT or OCT-HDT angle with the RNFL/GCIPL thickness for the nasal group remained true even after stratification. However, eyes with less severe myopia (SE ≥ − 6.00 D) showed more correlations between OI with the RNFL sectors (p2–p5) and GCIPL sectors (all sectors except ST).Table 5 shows the relationship between the myopic tilted disc parameters and the RNFL’s peak position and thickness. For the temporal group, a larger OI correlated with a smaller superior RNFL peak angle (β = − 43.73; CI: − 73.09 to − 14.37; *P* < 0.01) and a correspondingly smaller superior-inferior RNFL peak angle (β = − 52.79; CI: − 93.20 to − 12.38; *P* = 0.01). A larger temporal ODT angle correlated with a more significant superior-inferior RNFL peaks thickness asymmetry (β = 0.28; CI: 0.05 to 0.51; *P* = 0.02). For the nasal group, there was no significant association between the myopic tilted disc parameters and the position or thickness of the RNFL peak. In the above analyses, AL was adjusted instead of SE because axial elongation is related to myopic optic disc formation, influenced RNFL measurements [10], and is a known factor for changes in myopic ocular structure [27, 28].

**Table 4:** Relationship between optic disc morphology and OCT parameters—subgroup analysis

Sectors	Temporal group (n = 335)						Nasal group (n = 195)					
	Optic disc torsion angle (°)^a^		OCT horizontal tilt angle (°)^b^		Ovality index^c^		Optic disc torsion angle (°)^a^		OCT horizontal tilt angle (°)^b^		Ovality index^c^	
	Coefficient (95% CI)	*P* value	Coefficient (95% CI)	*P* value	Coefficient (95% CI)	*P* value	Coefficient (95% CI)	*P* value	Coefficient (95% CI)	*P* value	Coefficient (95% CI)	*P* value
Macular ganglion cell inner-plexiform layer (GCIPL) (μm)												
ST	0.01 (− 0.03, 0.05)	0.58	− 0.08 (− 0.16, − 0.01)	**0.04**	− 0.67 (− 5.57, 4.23)	0.79	0.02 (− 0.02, 0.05)	0.33	− 0.04 (− 0.11, 0.04)	0.32	− 4.35 (− 10.44, 1.75)	0.16
S	0.40 × 10^−2^ (− 0.03, 0.04)	0.81	− 0.07 (− 0.13, − 0.01)	**0.03**	− 1.90 (− 5.94, 2.14)	0.36	0.01 (− 0.03, 0.04)	0.79	− 0.01 (− 0.07, 0.06)	0.81	− 6.97 (− 13.04, − 0.91)	**0.02**
SN	0.10 × 10^−2^ (− 0.04, 0.04)	0.96	− 0.06 (− 0.14, 0.02)	0.14	− 3.24 (− 7.80, 1.33)	0.17	0.01 (− 0.22, 0.05)	0.51	− 0.03 (− 0.10, 0.04)	0.44	− 7.47 (− 13.95, − 0.98)	**0.02**
IT	0.02 (− 0.02, 0.06)	0.35	− 0.02 (− 0.11, 0.08)	0.76	− 2.72 (− 8.21, 2.79)	0.33	0.01 (− 0.03, 0.05)	0.52	− 0.04 (− 0.15, 0.07)	0.52	− 3.76 (− 12.52, 4.99)	0.40
I	− 0.10 × 10^−2^ (− 0.04, 0.03)	0.94	− 0.01 (− 0.09, 0.08)	0.90	− 3.06 (− 7.23, 1.11)	0.15	− 0.10 × 10^−2^ (− 0.04, 0.04)	0.95	− 0.03 (− 0.10, 0.05)	0.46	− 4.20 (− 10.39, 1.98)	0.18
IN	0.10 × 10^−2^ (− 0.04, 0.04)	0.96	− 0.06 (− 0.16, 0.04)	0.23	− 2.06 (− 6.97, 2.86)	0.41	0.30 × 10^−2^ (− 0.03, 0.04)	0.88	− 0.03 (− 0.13, 0.06)	0.49	− 1.94 (− 8.74, 4.86)	0.58
Peripapillary retinal nerve fiber layer (RNFL) (μm)												
p1	− 0.02 (− 0.17, 0.13)	0.79	− 0.49 (− 0.84, − 0.15)	**< 0.01**	− 50.26 (− 71.63, − 28.88)	**< 0.01**	0.13 (− 0.06, 0.31)	0.19	− 0.31 (− 0.71, 0.10)	0.14	− 33.31 (− 70.53, 3.91)	0.08
p2	0.11 (− 0.01, 0.23)	0.05	− 0.30 (− 0.58, − 0.03)	**0.03**	− 30.55 (− 47.93, − 13.17)	**< 0.01**	0.07 (− 0.11, 0.25)	0.42	− 0.25 (− 0.56, 0.06)	0.11	− 35.25 (− 61.46, − 9.05)	**0.01**
p3	0.04 (− 0.04, 0.13)	0.34	− 0.22 (− 0.42, − 0.03)	**0.02**	− 22.09 (− 36.82, − 7.37)	**< 0.01**	0.07 (− 0.06, 0.20)	0.27	0.02 (− 0.25, 0.28)	0.91	− 27.75 (− 49.81, − 5.69)	**0.01**
p4	0.09 (− 0.01, 0.20)	0.08	− 0.24 (− 0.47, − 0.02)	**0.04**	− 30.98 (− 43.26, − 18.70)	**< 0.01**	0.13 (− 0.01, 0.26)	0.08	− 0.04 (− 0.27, 0.20)	0.76	− 36.95 (− 61.03, − 12.88)	**< 0.01**
p5	0.12 (− 0.05, 0.28)	0.17	− 0.45 (− 0.83, − 0.08)	**0.02**	− 30.86 (− 52.69, − 9.03)	**< 0.01**	0.03 (− 0.23, 0.29)	0.84	− 0.17 (− 0.55, 0.21)	0.39	− 49.60 (− 82.72, − 16.48)	**< 0.01**
p6	0.13 (− 0.06, 0.32)	0.17	− 0.34 (− 0.77, 0.08)	0.11	− 8.76 (− 32.67, 15.16)	0.47	0.06 (− 0.21, 0.34)	0.65	− 0.20 (− 0.65, 0.26)	0.39	− 30.37 (− 72.31, 11.57)	0.16
p7	− 0.18 (− 0.35, − 0.01)	**0.04**	− 0.17 (− 0.63, 0.29)	0.46	22.39 (− 1.93, 46.71)	0.07	− 0.08 (− 0.32, 0.17)	0.54	0.07 (− 0.53, 0.66)	0.82	12.43 (− 33.82, 58.67)	0.60
p8	− 0.18 (− 0.32, − 0.04)	**0.01**	− 0.18 (− 0.52, 0.17)	0.32	29.02 (9.81, 48.22)	**< 0.01**	− 0.14 (− 0.35, 0.07)	0.18	− 0.11 (− 0.53, 0.30)	0.60	45.73 (1.06, 90.39)	0.05
p9	− 0.05 (− 0.16, 0.06)	0.40	− 0.26 (− 0.51, − 0.01)	0.05	34.57 (18.52, 50.61)	**< 0.01**	− 0.08 (− 0.19, 0.03)	0.15	0.2 × 10^−2^ (− 0.21, 0.21)	0.99	16.98 (− 6.49, 40.44)	0.16
p10	− 0.06 (− 0.23, 0.11)	0.48	− 0.14 (− 0.54, 0.25)	0.48	56.89 (33.37, 80.41)	**< 0.01**	− 0.16 (− 0.33, 0.01)	0.06	0.02 (− 0.35, 0.39)	0.91	14.94 (− 24.00, 53.87)	0.45
p11	− 0.16 (− 0.32, 0.01)	0.06	− 0.23 (− 0.65, 0.18)	0.27	1.73 (− 19.49, 22.94)	0.87	− 0.14 (− 0.30, 0.02)	0.10	− 0.08 (− 0.49, 0.34)	0.73	3.53 (− 35.62, 42.68)	0.86
p12	− 0.09 (− 0.31, 0.12)	0.40	− 0.56 (− 0.95, − 0.17)	**< 0.01**	− 29.46 (− 50.49, − 8.43)	**< 0.01**	0.07 (− 0.13, 0.26)	0.51	− 0.19 (− 0.62, 0.24)	0.38	8.60 (− 25.88, 43.09)	0.63

*OCT* = optical coherence tomography; *CI* = confidence interval; *SN* = superonasal; *IN* = inferonasal; *I* = inferior; *IT* = inferotemporal; *ST* = superotemporal; *S* = superior

The p1–p12 represent the 12 segmentations of RNFL in clockwise and anti-clockwise directions for the right and left eye, respectively

Generalized estimation equation adjusted for age, axial length and the other myopic tilted disc parameters (i.e., additionally adjusted for ^a^OCT horizontal tilt angle and ovality index, ^b^optic disc torsion angle and ovality index, and ^c^optic disc torsion angle and OCT horizontal tilt angle)

*P* values in bold indicate statistical significance

**Table 5:** Relationship between the myopic tilted disc parameters and retinal nerve fiber layer peak position and peak thickness

Variables	Optic disc morphology vs. peripapillary RNFL peak position					
	Superior RNFL peak angle (°)		Inferior RNFL peak angle (°)		Superior-inferior RNFL peak angle (°)	
	Coefficient (95% CI)	*P* value	Coefficient (95% CI)	*P* value	Coefficient (95% CI)	*P* value
Nasal group						
Optic disc torsion angle (°)^a^	0.04 (− 0.16, 0.24)	0.69	0.03 (− 0.15, 0.20)	0.78	0.07 (− 0.26, 0.39)	0.69
OCT horizontal tilt angle (°)^b^	0.07 (− 0.56, 0.71)	0.82	0.12 (− 0.28, 0.51)	0.56	0.19 (− 0.68, 1.06)	0.67
Ovality index^c^	− 8.45 (− 48.60, 31.70)	0.68	− 13.13 (− 37.42, 11.16)	0.29	− 21.57 (− 64.50, 21.36)	0.33
Temporal group						
Optic disc torsion angle (°)^a^	0.09 (− 0.09, 0.27)	0.32	0.11 (− 0.06, 0.28)	0.19	0.20 (− 0.07, 0.47)	0.14
OCT horizontal tilt angle (°)^b^	− 0.34 (− 0.81, 0.12)	0.15	0.10 (− 0.27, 0.47)	0.58	− 0.24 (− 0.96, 0.48)	0.51
Ovality index^c^	− 43.73 (− 73.09, − 14.37)	**< 0.01**	− 9.06 (− 32.09, 13.97)	0.44	− 52.79 (− 93.20, − 12.38)	**0.01**

Variables	Optic disc morphology vs. peripapillary RNFL peak thickness					
	Superior RNFL peak thickness (μm)		Inferior RNFL peak thickness (μm)		Superior-inferior RNFL peaks thickness asymmetry (μm)	
	Coefficient (95% CI)	*P* value	Coefficient (95% CI)	*P* value	Coefficient (95% CI)	*P* value
Nasal group						
Optic disc torsion angle (°)^a^	− 0.09 (− 0.39, 0.21)	0.56	− 0.28 (− 0.73, 0.16)	0.21	0.19 (− 0.28, 0.67)	0.43
OCT horizontal tilt angle (°)^b^	− 0.09 (− 0.71, 0.54)	0.79	− 0.268 (− 1.31, 0.78)	0.62	0.18 (− 0.53, 0.89)	0.61
Ovality index^c^	− 25.37 (− 67.93, 17.19)	0.24	− 55.14 (− 112.57, 2.29)	0.06	29.77 (− 18.14, 77.69)	0.22
Temporal group						
Optic disc torsion angle (°)^a^	0.14 (− 0.07, 0.35)	0.19	− 0.09 (− 0.36, 0.18)	0.50	0.28 (0.05, 0.51)	**0.02**
OCT horizontal tilt angle (°)^b^	− 0.70 (− 1.43, 0.04)	0.06	− 0.35 (− 1.46, 0.76)	0.54	− 0.44 (− 1.08, 0.20)	0.18
Ovality index^c^	− 5.56 (− 46.12, 35.00)	0.79	6.96 (− 48.07, 61.99)	0.80	− 3.92 (− 45.80, 37.96)	0.85

*RNFL* = retinal nerve fiber layer; *CI* = confidence interval

Generalized estimation equation adjusted for age, axial length and the other myopic tilted disc parameters (i.e., additionally adjusted for ^a^OCT horizontal tilt angle and ovality index, ^b^optic disc torsion angle and ovality index, and ^c^optic disc torsion angle and OCT horizontal tilt angle)

*P* values in bold indicate statistical significance

The interobserver reproducibility showed good reliability in measurement of OI, optic ODT angle, OCT-HDT angle, and angle of the superior and inferior RNFL peak (ICC of 0.82, 0.88, 0.88, 0.90, and 0.89, respectively) [25].

## Discussion

With AL and age adjusted, this study demonstrated that myopic optic disc changes were associated with different RNFL and GCIPL sectors. Based on the findings of different characteristics between eyes with different ODT directions and evidence supporting their diverse scleral deformation [18], this study divided the eyes into temporal and nasal groups and found different correlation patterns. Sung et al. evaluated 220 right eyes of young, healthy subjects with a relatively modest magnitude of myopia (mean age: 27.94 ± 6.67 years, SE: − 4.58 ± 2.66 D, and AL: 25.8 ± 1.5 mm). The authors showed that eyes with counterclockwise-torted (temporally-torted) optic discs had longer AL, more myopic SE, larger values of myopic optic disc parameters (equivalent to OI and ODT of the current study), thinner inferonasal GCIPL, and thinner superior and inferior RNFL than eyes with clockwise-torted (nasally-torted) optic disc [26]. This study included 530 eyes (left and right) of older-aged, healthy participants with a larger magnitude of myopia (mean age: 41.7 ± 5.8 years, SE: − 7.70 ± 3.11 D and AL: 26.6 ± 1.4 mm). Our current study demonstrated similar findings (statistically longer AL, more myopic SE, larger ODT angle, OCT-HDT angle, and OI of the eyes with temporal ODT) but different patterns of RNFL/GCIPL thickness. After adjusting for AL and age, the two groups had similar inferior RNFL thickness (this finding was in contrast to Sung’s study [26]). Similarly, we found a thinner superior RNFL and inferonasal GCIPL, the temporal group had thinner inferior GCIPL, thinner nasal RNFL, and thicker temporal RNFL (Table 2); the latter was also noted in other studies [16, 17]. The discrepancies could be attributed to the different study population. Our study has a smaller ODT angle (23.53 ± 20.38° vs. 28.93 ± 26.55° in Sung et al. [26]), which may explain the insignificant difference in the inferior RNFL thickness, since a larger ODT angle correlated with thinner p7–8 RNFL.

The temporal group showed more associations between the myopic optic disc parameters and the RNFL/GCIPL thickness than the nasal group. For the temporal group, a larger ODT angle correlated with thinner inferonasal (p7–8) RNFL and a larger superior-inferior RNFL peak thickness asymmetry. A larger OCT-HDT angle correlated with thinner nasal (p12–5) RNFL and thinner superior-to-superonasal GCIPL. Such correlations became more apparent in eyes with SE ≥  − 6.00 D compared with severe myopia based on our subgroup analysis (Supplementary Table 3) in eyes with severe myopia. The thinner GCIPL may result from increased peripapillary tissue strain due to disc tilting, as reflected by a previous study showing the association between increased disc tilt and wider peripapillary crescent width [29]. Such associations did not exist in the nasal group. Another study also showed the association between a smaller angle of inferotemporal peak and a larger ODT angle [30]. However, after dividing the myopic eyes into the temporal and nasal groups, we did not find such an association.

In contrast, OI was associated with RNFL in both groups. For the temporal group, an increased OI correlated with a closer distance between the superior RNFL peak and the fovea (i.e., a small superior RNFL peak angle and superior-inferior RNFL peak angle). An increase OI also correlated with thinner nasal RNFL and thicker temporal RNFL (which remained true after the myopia severity subgroup analysis), consistent with previous studies [14, 16] and reflecting that RNFL at p12–5 were stretched and rotated superotemporally towards the p8–10 region. For the nasal group, increased OI was also associated with thinner nasal (p2–5) RNFL (with only a borderline association with a thicker p8 RNFL) and thinner superior-to-superonasal GCIPL. The association was more apparent in eyes with SE ≥ − 6.00 D. The contrasting results could be related to the nasal group’s smaller sample size, OI value, ODT angle, and OCT-HDT angle (Table 2). It could also be related to their possible underlying diverse myopization mechanisms.

Myopic structural changes reduced the accuracy of OCT measurements, challenging the diagnostic accuracy of myopic glaucoma [6–10]. Although several myopic eye normative databases were built based on AL or SE [11, 31–34], the results showed that OI, ODT, and OCT-HDT angles correlated with different RNFL/GCIPL sectors independent of age and AL, with different ODT directions showing different association patterns. Establishing specific normative RNFL/GCIPL data for different optic disc morphological changes could improve the accuracy of the database (e.g., separate normative RNFL and GCIPL database for eyes with different directions of ODT). This is essential since integral analysis of RNFL and GCIPL improved the detection of pre-perimetric and early glaucoma [19].

The observation of this study also provides critical information for myopic disc development; several mechanisms have been proposed [12, 18, 35, 36]. It was suggested that during myopization, the BMO remains relatively unchanged while the lamina cribrosa and peripapillary sclera shift dramatically and rotate the ganglion cell axons through the BMO window [35, 36]. The scleral thickness is also altered and the weakest point becomes susceptible to DPE formation, generating an additional force that pulls the lamina cribrosa backward, causing the optic disc to tilt around the vertical axis [12, 18] and an oval-shaped optic disc with an elevated margin at the shifting direction of the lamina cribrosa. The findings of different ODT directions having different association patterns between the myopic tilted disc parameters and RFNL/GCIPL sectors suggested that temporal and nasal ODT may have different myopization mechanisms. Eyes with temporal ODT have more RNFL and GCIPL sectors correlated with the OCT-HDT angle than the ODT angle, reflecting the involvement of the DPE pulling force. In contrast, only OI was associated with the RNFL and GCIPL sectors in eyes with nasal ODT, suggesting that the formation of myopic titled disc was prominently affected by the disc deformation. However, prospective studies are needed to verify the possible mechanisms.

This study has several strengths, including large sample size (530 eyes), involvement of left and right eyes, a large proportion of high myopia (92% of eyes had SE < − 6.00 D), and stratified analyses of nasal and temporal ODT eyes that showed contrasting implications between the two groups. The participants’ average age of 41.7 years old reflect a more precise evaluation of the 40-year-old myopic group, an age group known for its increasing prevalence of primary open-angle glaucoma. However, this study also has limitations. First, this study only involved Asian participants and may not apply to other ethnic groups. Second, this is a cross-sectional study; future longitudinal studies may help reveal the change in progressive optic disc morphology and its dynamic effect on RNFL and GCIPL thickness. Third, the variable size of peripapillary atrophy could lead to the circle scan misaligning and may introduce errors in the measurement. Fourth, astigmatism may also introduce a magnification effect in the study, resulting in a more oval appearance of the optic disc, and an inaccurate displacement of the scanning circle from the optic disc, leading to inaccurate measurement of the RNFL thickness [37, 38]. To limit the measurement variability, this study employed two readers who demonstrated good reliability. Fifth, we did not use contact lens during OCT imaging when we encountered difficult situations (e.g., eyes with posterior staphyloma). Inclusion of the contact lens may have enhanced the image quality and increased our sample size [39].

## Conclusion

In conclusion, temporal torsion and nasal torsion showed different patterns of association between myopic tilted disc parameters (OI, OCT HDT, and ODT angle) and RNFL/GCIPL thickness. When evaluating OCT images of eye with myopic tilted disc, this impact of myopic tilted disc should be considered before diagnosing myopic glaucoma.

## Supplementary Information

Supplementary Material 1

## Data Availability

The data and materials that support the findings of this study are available from the corresponding author upon reasonable request.
