# Supplementary material for: Exploring optical coherence tomography parameters in eyes with myopic tilted disc
Source: Eye Vis (Lond). 2024 Nov 2;11:47. doi: 10.1186/s40662-024-00411-3 (PMC11580533; doi:10.1186/s40662-024-00411-3)
Supplement: Supplementary file 1 — Supplementary Material 1 [file 40662_2024_411_MOESM1_ESM.docx]

**Supplementary** **Table 1.** Relationship between optic disc morphology and OCT parameters (adjusted by spherical equivalent instead of axial length).

|  | **Temporal group (n=335)** | | | | | |  | **Nasal group (n=195)** | | | | | |
| --- | --- | --- | --- | --- | --- | --- | --- | --- | --- | --- | --- | --- | --- |
|  | **Optic disc torsion**  **angle (°)^a^** | | **OCT horizontal tilt**  **angle (°)^b^** | | **Ovality index^c^** | |  | **Optic disc torsion**  **angle (°)^a^** | | **OCT horizontal tilt**  **angle (°)^b^** | | **Ovality index^c^** | |
| **Sectors** | **Coefficient**  **(95% CI)** | ***P* value** | **Coefficient**  **(95% CI)** | ***P* value** | **Coefficient**  **(95% CI)** | ***P* value** |  | **Coefficient**  **(95% CI)** | ***P* value** | **Coefficient (95% CI)** | ***P* value** | **Coefficient**  **(95% CI)** | ***P* value** |
| Macular ganglion cell inner-plexiform layer (GCIPL) (μm) | | | | | | | | | | | | | |
| ST | 0.50×10^−2^  (−0.03, 0.04) | 0.79 | −0.08  (−0.15, −0.01) | **0.03** | −0.21  (−4.95, 4.54) | 0.93 |  | 0.02  (−0.02, 0.05) | 0.32 | −0.03  (−0.11, 0.04) | 0.36 | −2.68  (−8.46, 3.10) | 0.36 |
| S | −0.40×10^−2^  (−0.03, 0.03) | 0.80 | −0.07  (−0.13, −0.01) | **0.03** | −1.41  (−5.40, 2.57) | 0.49 |  | 0.20×10^−2^  (−0.03, 0.04) | 0.90 | −0.01  (−0.08, 0.05) | 0.68 | −4.37  (−10.15, −1.42) | 0.14 |
| SN | −0.90×10^−2^  (−0.04, 0.03) | 0.62 | −0.07  (−0.14, 0.01) | 0.09 | −2.39  (−6.48, 1.98) | 0.28 |  | 0.01  (−0.03, 0.04) | 0.60 | −0.04  (−0.11, 0.04) | 0.33 | −4.63  (−10.94, 1.68) | 0.15 |
| IT | 0.01  (−0.03, 0.05) | 0.52 | −0.01  (−0.10, 0.08) | 0.77 | −2.19  (−7.53, 3.16) | 0.42 |  | 0.01  (−0.03, 0.05) | 0.50 | −0.03  (−0.14, 0.08) | 0.59 | −1.77  (−9.99, 6.44) | 0.67 |
| I | −0.01  (−0.04, 0.02) | 0.52 | −0.01  (−0.09, 0.06) | 0.78 | −2.23  (−6.29, 1.84) | 0.28 |  | −0.30×10^−2^  (−0.04, 0.03) | 0.86 | −0.03  (−0.11, 0.04) | 0.38 | −1.04  (−6.73, 4.65) | 0.72 |
| IN | −0.01  (−0.05, 0.03) | 0.59 | −0.07  (−0.16, 0.03) | 0.15 | −1.12  (−5.76, 3.52) | 0.64 |  | 0.10×10^−2^  (−0.03, 0.04) | 0.94 | −0.03  (−0.13, 0.06) | 0.45 | 0.76  (−5.49, 7.00) | 0.81 |
| Peripapillary retinal nerve fiber layer (RNFL) (μm) | | | | | | | | | | | | | |
| p1 | −0.04  (−0.19, 0.11) | 0.59 | −0.59  (−0.96, −0.21) | **<0.01** | −45.38  (−67.61, −23.60) | **<0.01** |  | 0.13  (−0.06, 0.33) | 0.17 | −0.33  (−0.73, 0.06) | 0.10 | −23.99  (−61.03, 13.05) | 0.20 |
| p2 | 0.10  (−0.01, 0.21) | 0.09 | −0.34  (−0.60, −0.08) | **0.01** | −29.44  (−46.59, −12.29) | **<0.01** |  | 0.08  (−0.09, 0.26) | 0.36 | −0.20  (−0.50, 0.10) | 0.19 | −31.37  (−56.23, −6.50) | **0.01** |
| p3 | 0.03  (−0.06, 0.12) | 0.50 | −0.18  (−0.37, −0.02) | 0.07 | −23.68  (−38.49, −8.87) | **<0.01** |  | 0.06  (−0.06, 0.18) | 0.35 | 0.80×10^−2^  (−0.25, 0.27) | 0.95 | −25.25  (−45.89, −4.62) | **0.02** |
| p4 | 0.07  (−0.04, 0.18) | 0.20 | −0.21  (−0.41, 0.10×10^−2^) | 0.05 | −31.51  (−43.81, −19.21) | **<0.01** |  | 0.12  (−0.02, 0.25) | 0.10 | −0.04  (−0.28, 0.20) | 0.75 | −32.06  (−55.00., −9.13) | **0.01** |
| p5 | 0.09  (−0.08, 0.26) | 0.31 | −0.35  (−0.74, 0.03) | 0.07 | −34.73  (−56.83, −12.63) | **<0.01** |  | −0.40×10^−2^  (−0.26, 0.25) | 0.98 | −0.27  (−0.69, 0.14) | 0.20 | −33.22  (−65.81, −0.63) | **0.05** |
| p6 | 0.09  (−0.11, 0.28) | 0.38 | −0.30  (−0.71, 0.11) | 0.15 | −8.77  (−32.70,15.15) | 0.47 |  | 0.80x10^−2^  (−0.27, 0.28) | 0.96 | −0.33  (−0.81, 0.15) | 0.18 | −5.61  (−47.70, 36.47) | 0.79 |
| p7 | −0.20  (−0.38, −0.03) | **0.02** | −0.30  (−0.80, 0.20) | 0.24 | 30.06  (4.70, 55.43) | **0.02** |  | −0.08  (−0.33, 0.17) | 0.52 | 0.03  (−0.56, 0.62) | 0.92 | 17.97  (−26.42, 62.37) | 0.43 |
| p8 | −0.17  (−0.31, −0.03) | **0.02** | −0.25  (−0.60, 0.10) | 0.16 | 31.50  (12.48, 50.53) | **<0.01** |  | −0.12  (−0.33, 0.08) | 0.24 | −0.06  (−0.50, 0.38) | 0.80 | 39.07  (−5.60, 83.73) | 0.09 |
| p9 | −0.05  (−0.16, 0.06) | 0.37 | −0.27  (−0.51, −0.04) | **0.02** | 35.18  (19.10, 51.26) | **<0.01** |  | −0.07  (−0.18, 0.03) | 0.19 | 0.03  (−0.20, 0.25) | 0.82 | 15.25  (−7.76, 38.27) | 0.19 |
| p10 | −0.07  (−0.23, 0.10) | 0.43 | 0.15  (−0.53, 0.22) | 0.42 | 57.73  (34.36, 81.09) | **<0.01** |  | −0.16  (−0.33, 0.10×10^−2^) | 0.05 | 0.03  (−0.36, 0.42) | 0.89 | 13.55  (−24.62, 51.72) | 0.49 |
| p11 | −0.17  (−0.33, −0.10×10^−2^) | **0.05** | −0.29  (−0.69, 0.12) | 0.16 | 6.44  (−14.35, 27.24) | 0.54 |  | −0.15  (−0.32, 0.03) | 0.09 | −0.11  (−0.56, 0.34) | 0.62 | 9.20  (−29.37, 47.77) | 0.64 |
| p12 | −0.09  (−0.31, 0.13) | 0.40 | −0.62  (−1.00, −0.24) | **<0.01** | −23.07  (−44.28, −1.87) | **0.03** |  | 0.07  (−0.14, 0.27) | 0.51 | −0.27  (−0.71, 0.18) | 0.25 | 20.80  (−12.87, 54.47) | 0.23 |

The p1–p12 represent the 12 segmentations of RNFL in clockwise and anti-clockwise directions for the right and left eye, respectively.

OCT = optical coherence tomography; CI = confidence interval; SN = superonasal; IN = inferonasal; I = inferior; IT = inferotemporal; ST = superotemporal; S = superior.

Generalized estimation equation adjusted for age, axial length and the other myopic tilted disc parameters (i.e., additionally adjusted for, ^a^OCT horizontal tilt angle and ovality index, ^b^optic disc torsion angle and ovality index, and ^c^optic disc torsion angle and OCT horizontal tilt angle).

*P* values in bold indicate statistical significance.

**Supplementary Table 2.** Relationship between optic disc morphology and RNFL peak position and thickness (adjusted by spherical equivalent instead of axial length).

|  | **Optic disc morphology vs. peripapillary RNFL peak position** | | | | | | | | | |  |  |
| --- | --- | --- | --- | --- | --- | --- | --- | --- | --- | --- | --- | --- |
|  | **Superior RNFL peak angle (°)** | |  | **Inferior RNFL peak angle (°)** | |  | **Angle between superior and inferior RNFL peak (°)** | | | | |  |
| **Variables** | **Coefficient**  **(95% CI)** | ***P* value** |  | **Coefficient**  **(95% CI)** | ***P* value** |  | **Coefficient**  **(95% CI)** | | ***P* value** | | |  |
| Nasal group | | | | | | | | | | |  |  |
| Optic disc torsion angle (°)^a^ | 0.05  (−0.16, 0.26) | 0.63 |  | 0.04  (−0.14, 0.22) | 0.66 |  | | 0.09  (−0.25, 0.44) | | 0.60 | | |
| OCT horizontal tilt angle (°)^b^ | 0.03  (−0.60, 0.66) | 0.93 |  | 0.09  (−0.29, 0.47) | 0.65 |  | | 0.12  (−0.76, 0.99) | | 0.80 | | |
| Ovality index^c^ | −5.56  (−44.65, 33.53) | 0.78 |  | −8.10  (−30.52, 14.32) | 0.48 |  | | −13.65  (−56.22, 28.92) | | 0.53 | | |
| Temporal group | | | | | | | | | | |  |  |
| Optic disc torsion angle (°)^a^ | 0.10  (−0.08, 0.29) | 0.28 |  | 0.11  (−0.06, 0.27) | 0.20 |  | | 0.21  (−0.06, 0.48) | | 0.13 | | |
| OCT horizontal tilt angle (°)^b^ | −0.38  (−0.83, −0.07) | 0.10 |  | 0.08  (−0.27, 0.43) | 0.66 |  | | −0.30  (−0.97, 0.37) | | 0.38 | | |
| Ovality index^c^ | −34.85  (−63.62, −6.09) | **0.02** |  | −6.25  (−29.90, 17.39) | 0.60 |  | | −41.10  (−79.36, −2.84) | | **0.04** | | |
|  | **Optic disc morphology vs. peripapillary RNFL peak thickness** | | | | | | | | | |  |  |
|  | **Superior RNFL peak thickness (μm)** | |  | **Inferior RNFL peak thickness (μm)** | |  | | **Asymmetry between superior and inferior RNFL peak (μm)** | | | | |
| **Variables** | **Coefficient**  **(95% CI)** | ***P* value** |  | **Coefficient**  **(95% CI)** | ***P* value** |  | | **Coefficient**  **(95% CI)** | | ***P* value** | | |
| Nasal group | | | | | | | | | | |  |  |
| Optic disc torsion angle (°)^a^ | −0.06  (−0.36, 0.24) | 0.68 |  | −0.24  (−0.66, 0.19) | 0.27 |  | | 0.18  (−0.29, 0.64) | | 0.46 | | |
| OCT horizontal tilt angle (°)^b^ | −0.13  (−0.77, 0.51) | 0.69 |  | −0.36  (−1.38, 0.66) | 0.49 |  | | 0.23  (−0.50, 0.96) | | 0.53 | | |
| Ovality index^c^ | −16.18  (−58.74, 26.38) | 0.46 |  | −40.65  (−96.78, 15.47) | 0.16 |  | | 24.47  (−23.03, 71.97) | | 0.31 | | |
| Temporal group | | | | | | | | | | |  |  |
| Optic disc torsion angle (°)^a^ | 0.12  (−0.08, 0.31) | 0.23 |  | −0.11  (−0.37, 0.14) | 0.38 |  | | 0.30  (0.04, 0.56) | | **0.03** | | |
| OCT horizontal tilt angle (°)^b^ | −0.74  (−1.47, −0.01) | **0.05** |  | −0.39  (−1.48, 0.70) | 0.48 |  | | −0.44  (−1.07, 0.18) | | 0.17 | | |
| Ovality index^c^ | −3.22  (−42.08, 35.64) | 0.87 |  | 9.88  (−42.93, 62.69) | 0.71 |  | | 0.50  (−49.14, 50.14) | | 0.98 | | |

RNFL = retinal nerve fiber layer; CI = confidence interval.

Generalized estimation equation adjusted for age, spherical equivalent, and the other myopic tilted disc parameters (i.e., additionally adjusted for ^a^OCT horizontal tilt angle and ovality index, ^b^optic disc torsion angle and ovality index, and ^c^optic disc torsion angle and OCT horizontal tilt angle).

*P* values in bold indicate statistical significance.

**Supplementary Table 3.** Relationship between optic disc morphology and OCT parameters based on severity of myopia – subgroup analysis for temporal group.

|  | **Spherical equivalent < −6.00 D** **(n=274)** | | | | | |  | **Spherical equivalent ≥ −6.00 D** **(n= 61)** | | | | | |
| --- | --- | --- | --- | --- | --- | --- | --- | --- | --- | --- | --- | --- | --- |
|  | **Optic disc torsion**  **angle (°)^a^** | | **OCT horizontal tilt**  **angle (°)^b^** | | **Ovality index^c^** | |  | **Optic disc torsion**  **angle (°)^a^** | | **OCT horizontal tilt**  **angle (°)^b^** | | **Ovality index^c^** | |
| **Sectors** | **Coefficient**  **(95% CI)** | ***P* value** | **Coefficient**  **(95% CI)** | ***P* value** | **Coefficient**  **(95% CI)** | ***P* value** |  | **Coefficient**  **(95% CI)** | ***P* value** | **Coefficient**  **(95% CI)** | ***P* value** | **Coefficient**  **(95% CI)** | ***P* value** |
| Macular ganglion cell inner-plexiform layer (GCIPL) (μm) | | | | | | | | | | | | | |
| ST | 0.01  (−0.03, 0.05) | 0.65 | −0.07  (−0.16, 0.02) | 0.13 | −0.48  (−5.73, 4.77) | 0.86 |  | 0.05  (−0.03, 0.12) | 0.23 | −0.21  (−0.39, −0.04) | **0.02** | 5.26  (−8.24, 18.75) | 0.45 |
| S | 0.01  (−0.03, 0.04) | 0.78 | −0.07  (−0.14, 0.40×10^−2^) | 0.07 | −1.53  (−5.86, 2.80) | 0.49 |  | 0.03  (−0.03, 0.08) | 0.34 | −0.10  (−0.23, 0.03) | 0.15 | −0.78  (−11.97, 10.41) | 0.89 |
| SN | 0.30×10^−2^  (−0.04, 0.04) | 0.90 | −0.06  (−0.15, 0.03) | 0.21 | −3.04  (−7.83, 1.75) | 0.21 |  | 0.01  (−0.07, 0.09) | 0.81 | −0.08  (−0.24, 0.07) | 0.29 | −0.99  (−15.03,13.05) | 0.89 |
| IT | 0.02  (−0.03, 0.07) | 0.43 | 0.01  (−0.10, 0.12) | 0.84 | −2.92  (−8.84, 2.99) | 0.33 |  | 0.05  (−0.03, 0.12) | 0.20 | −0.23  (−0.39, −0.06) | **0.01** | 7.83  (−4.98, 20.64) | 0.23 |
| I | −0.30×10^−2^  (−0.04, 0.04) | 0.86 | 0.02  (−0.07, 0.11) | 0.66 | −2.95  (−7.40, 1.50) | 0.19 |  | 0.01  (−0.07, 0.10) | 0.73 | −0.11  (−0.24, 0.02) | 0.10 | −0.19  (−10.73, 10.34) | 0.97 |
| IN | −0.01  (−0.05, 0.04) | 0.75 | −0.05  (−0.15, 0.06) | 0.39 | −2.38  (−7.52, 2.76) | 0.36 |  | 0.07  (−0.06, 0.19) | 0.32 | −0.11  (−0.25, 0.03) | 0.13 | 4.83  (−7.86, 17.53) | 0.46 |
| Peripapillary retinal nerve fiber layer (RNFL) (μm) | | | | | | | | | | | | | |
| p1 | −0.01  (−0.18, 0.15) | 0.88 | −0.44  (−0.81, −0.07) | **0.02** | −48.17  (−70.44, −25.90) | **<0.01** |  | 0.02  (−0.26, 0.30) | 0.90 | −0.12  (−1.17, 0.93) | 0.82 | −45.39  (−82.23, −8.55) | **0.02** |
| p2 | 0.10  (−0.01, 0.22) | 0.08 | −0.18  (−0.45, 0.10) | 0.21 | −25.47  (−43.43, −7.51) | **<0.01** |  | 0.22  (0.11, 0.33) | **<0.01** | −0.29  (−0.57, −0.10×10^−2^) | **0.05** | −61.21  (−87.10, −35.32) | **<0.01** |
| p3 | 0.02  (−0.07, 0.10) | 0.70 | −0.16  (−0.37, 0.04) | 0.12 | −20.75  (−36.75, −4.76) | **0.01** |  | 0.26  (0.11, 0.40) | **<0.01** | −0.33  (−0.60, −0.05) | **0.02** | −29.80  (−49.69, −9.90) | **<0.01** |
| p4 | 0.10  (−0.01, 0.20) | 0.07 | −0.13  (−0.34, 0.08) | 0.23 | −27.23  (−39.64, −14.82) | **<0.01** |  | 0.09  (−0.15, 0.32) | 0.47 | −0.30  (−0.70, 0.10) | 0.15 | −43.23  (−63.16, −23.30) | **<0.01** |
| p5 | 0.13  (−0.05, 0.30) | 0.15 | −0.44  (−0.82, −0.07) | **0.02** | −27.67  (−50.33, −5.01) | **0.02** |  | 0.01  (−0.23, 0.25) | 0.94 | 0.84  (0.06, 1.61) | **0.03** | −69.63  (−135.76, −3.49) | **0.03** |
| p6 | 0.15  (−0.05, 0.35) | 0.14 | −0.40  (−0.84, 0.04) | 0.07 | −8.05  (−33.63, 17.54) | 0.54 |  | −0.05  (−0.40, 0.29) | 0.76 | 1.45  (0.16, 2.74) | **0.03** | −36.83  (−99.87, 26.21) | 0.25 |
| p7 | −0.20  (−0.39, −0.02) | **0.03** | −0.25  (−0.78, 0.28) | 0.36 | 13.51  (−10.62, 37.63) | 0.27 |  | 0.03  (−0.23, 0.28) | 0.83 | −0.42  (−1.47, 0.63) | 0.43 | 110.36  (58.45, 162.27) | **<0.01** |
| p8 | −0.19  (−0.34, −0.04) | **0.02** | −0.20  (−0.59, 0.19) | 0.31 | 23.67  (3.97, 43.36) | **0.02** |  | −0.07  (−0.24, 0.11) | 0.47 | −0.64  (−1.44, 0.15) | 0.11 | 95.12  (48.23, 142.01) | **<0.01** |
| p9 | −0.02  (−0.14, 0.10) | 0.73 | −0.36  (−0.64, −0.08) | **0.01** | 33.01  (16.02, 49.99) | **<0.01** |  | −0.14  (−0.25, −0.03) | **0.01** | 0.35  (−0.23, 0.92) | 0.23 | 51.89  (29.77, 74.02) | **<0.01** |
| p10 | −0.03  (−0.21, 0.15) | 0.73 | −0.28  (−0.74, 0.17) | 0.22 | 53.76  (28.53, 78.99) | **<0.01** |  | −0.13  (−0.34, 0.09) | 0.26 | 0.27  (−0.89, 1.43) | 0.65 | 93.32  (49.07, 137.57) | **<0.01** |
| p11 | −0.16  (−0.35, 0.02) | 0.08 | −0.23  (−0.69, 0.23) | 0.34 | −2.51  (−25.22, 20.21) | 0.83 |  | −0.04  (−0.27, 0.18) | 0.70 | −1.17  (−2.07, −0.26) | **0.01** | 70.47  (13.28, 127.66) | **0.02** |
| p12 | −0.10  (−0.34, 0.14) | 0.42 | −0.37  (−0.80, 0.07) | 0.10 | −30.75  (−52.98, −8.52) | **<0.01** |  | −0.12  (−0.55, 0.32) | 0.61 | −1.67  (−2.30, −1.04) | **<0.01** | 23.80  (−36.84, 84.45) | 0.44 |

The p1-p12 represent the 12 segmentations of RNFL in clockwise and anti-clockwise directions for the right and left eye, respectively.

OCT = optical coherence tomography; CI = confidence interval; SN = superonasal; IN = inferonasal; I = inferior; IT = inferotemporal; ST = superotemporal; S = superior.

Generalized estimation equation adjusted for age, axial length and the other myopic tilted disc parameters (i.e., additionally adjusted for ^a^OCT horizontal tilt angle and ovality index, ^b^optic disc torsion angle and ovality index, and ^c^optic disc torsion angle and OCT horizontal tilt angle).

*P* values in bold indicate statistical significance.

**Supplementary Table 4.** Relationship between optic disc morphology and OCT parameters based on severity of myopia – subgroup analysis for nasal group.

|  | **Spherical equivalent < −6.00 D (n = 159)** | | | | | |  | **Spherical equivalent ≥ −6.00 D (n = 36)** | | | | | |
| --- | --- | --- | --- | --- | --- | --- | --- | --- | --- | --- | --- | --- | --- |
|  | **Optic disc torsion**  **angle (°)^a^** | | **OCT horizontal tilt**  **angle (°)^b^** | | **Ovality index^c^** | |  | **Optic disc torsion**  **angle (°)^a^** | | **OCT horizontal tilt**  **angle (°)^b^** | | **Ovality index^c^** | |
| **Sectors** | **Coefficient (95% CI)** | ***P* value** | **Coefficient (95% CI)** | ***P* value** | **Coefficient**  **(95% CI)** | ***P* value** |  | **Coefficient (95% CI)** | ***P* value** | **Coefficient**  **(95% CI)** | ***P* value** | **Coefficient**  **(95% CI)** | ***P* value** |
| Macular ganglion cell inner-plexiform layer (GCIPL) (μm) | | | | | | | | | | | | | |
| ST | 0.02  (−0.02, 0.06) | 0.32 | −0.04  (−0.12, 0.043) | 0.35 | −2.92  (−9.64, 3.80) | 0.39 |  | 0.04  (−0.06, 0.15) | 0.41 | 0.08  (−0.07, 0.24) | 0.30 | −11.07  (−23.62, 1.48) | 0.08 |
| S | 0.01  (−0.03, 0.04) | 0.76 | −0.02  (−0.09, 0.05) | 0.76 | −5.38  (−12.34, 1.58) | 0.13 |  | 0.03  (−0.06, 0.12) | 0.54 | 0.11  (−0.05, 0.27) | 0.18 | −20.98  (−33.47, −8.48) | **<0.01** |
| SN | 0.01  (−0.02, 0.05) | 0.50 | −0.05  (−0.12, 0.02) | 0.18 | −6.649  (−13.85, 0.55) | 0.07 |  | 0.02  (−0.08, 0.11) | 0.75 | 0.17  (0.10×10^−2^, 0.33) | **0.05** | −20.25  (−32.02, −8.48) | **<0.01** |
| IT | 0.01  (−0.03, 0.06) | 0.58 | 0.03  (−0.15, 0.09) | 0.59 | −1.05  (−10.38, 8.28) | 0.83 |  | 0.04  (−0.08, 0.16) | 0.53 | 0.11  (−0.05, 0.27) | 0.17 | −16.31  (−30.95, −1.66) | **0.03** |
| I | −0.01  (−0.05, 0.04) | 0.79 | −0.03  (−0.11, 0.05) | 0.49 | −2.01  (−8.29, 4.28) | 0.53 |  | −0.20×10^−2^  (−0.09, 0.09) | 0.96 | 0.08  (−0.11, 0.27) | 0.41 | −15.96  (−30.08, −1.85) | **0.03** |
| IN | −0.30×10^−2^  (−0.043, 0.036) | 0.87 | −0.05  (−0.15, 0.06) | 0.38 | 0.21  (−7.00, 7.42) | 0.95 |  | 0.03  (−0.04, 0.11) | 0.39 | 0.15  (−0.01, 0.31) | 0.06 | −15.00  (−26.382 −3.62) | **0.01** |
| Peripapillary retinal nerve fiber layer (RNFL) (μm) | | | | | | | | | | | | | |
| p1 | 0.16  (−0.07, 0.39) | 0.17 | −0.20  (−0.60, 0.21) | 0.34 | −21.53  (−59.52, 16.45) | 0.27 |  | −0.35  (−0.79, 0.09) | 0.12 | −0.25  (−1.87, 1.37) | 0.76 | −75.88  (−165.27, 13.51) | 0.10 |
| p2 | 0.08  (−0.12, 0.27) | 0.44 | −0.10  (−0.41, 0.21) | 0.53 | −21.26  (−48.20, 5.68) | 0.12 |  | −0.10  (−0.44, 0.23) | 0.54 | −0.25  (−0.98, 0.47) | 0.49 | −60.56  (−112.64, −8.49) | **0.02** |
| p3 | 0.04  (−0.10, 0.19) | 0.57 | 0.07  (−0.20, 0.34) | 0.61 | −20.46  (−43.73, 2.80) | 0.09 |  | 0.08  (−0.14, 0.31) | 0.47 | 0.14  (−0.43, 0.70) | 0.63 | −41.71  (−76.92 −6.51) | **0.02** |
| p4 | 0.15  (−0.02, 0.31) | 0.08 | 0.07  (−0.17, 0.33) | 0.58 | −25.86  (−51.98, 0.27) | 0.05 |  | 0.01  (−0.22, 0.25) | 0.91 | −0.27  (−0.89, 0.34) | 0.38 | −80.50  (−114.72, −45.98) | **<0.01** |
| p5 | 0.05  (−0.24, 0.34) | 0.73 | −0.13  (−0.50, 0.24) | 0.48 | −40.21  (−74.73, −5.21) | **0.02** |  | −0.41  (−0.75, −0.07) | **0.02** | 0.55  (−0.63, 1.72) | 0.36 | −83.35  (−153.85, −12.86) | **0.02** |
| p6 | 0.06  (−0.25, 0.37) | 0.71 | −0.26  (−0.72, 0.21) | 0.28 | −27.13  (−69.04, 14.78) | 0.21 |  | −0.41  (−0.94, 0.12) | 0.13 | 0.94  (−0.27, 2.14) | 0.13 | −29.66  (−179.41, 120.09) | 0.70 |
| p7 | −0.09  (−0.37, 0.18) | 0.51 | 0.08  (−0.54, 0.70) | 0.79 | 3.03  (−45.06, 51.12) | 0.90 |  | 0.34  (−0.08, 0.76) | 0.11 | −0.87  (−2.53, 0.80) | 0.31 | 67.06  (−48.60, 182.72) | 0.26 |
| p8 | −0.18  (−0.42, 0.05) | 0.12 | −0.16  (−0.61, 0.29) | 0.49 | 45.35  (−2.57, 93.27) | 0.06 |  | 0.19  (−0.04, 0.42) | 0.11 | −0.23  (−1.39, 0.92) | 0.69 | 31.55  (−30.62, 93.72) | 0.32 |
| p9 | −0.10  (−0.23, 0.03) | 0.13 | −0.05  (−0.28, 0.18) | 0.67 | 17.45  (−7.85, 42.74) | 0.18 |  | −0.04  (−0.18, 0.10) | 0.55 | 0.20  (−0.63, 1.02) | 0.64 | 1.30  (−42.85, 45.45) | 0.95 |
| p10 | −0.20  (−0.38, −0.02) | **0.03** | −0.05  (−0.43, 0.32) | 0.78 | 10.32  (−30.70, 51.34) | 0.62 |  | 0.16  (−0.16, 0.49) | 0.32 | −0.13  (−1.53, 1.28) | 0.86 | 21.55  (−40.69, 83.79) | 0.50 |
| p11 | −0.15  (−0.32, 0.02) | 0.08 | −0.07  (−0.52, 0.37) | 0.75 | 0.91  (−42.27, 44.09) | 0.97 |  | −0.02  (−0.35, 0.32) | 0.93 | −0.67  (−1.34, 0.01) | 0.05 | 8.63  (−46.67, 63.93) | 0.76 |
| p12 | 0.06  (−0.19, 0.31) | 0.63 | −0.21  (−0.63, 0.21) | 0.33 | 15.39  (−20.11, 50.88) | 0.40 |  | −0.22  (−0.57, 0.13) | 0.23 | 0.35  (−1.43, 2.12) | 0.70 | −30.15  (−116.92, 56.62) | 0.50 |

The p1-p12 represent the 12 segmentations of RNFL in clockwise and anti-clockwise directions for the right and left eye, respectively.

OCT = optical coherence tomography; CI = confidence interval; SN = superonasal; IN = inferonasal; I = inferior; IT = inferotemporal; ST = superotemporal; S = superior.

Generalized estimation equation adjusted for age, axial length and the other myopic tilted disc parameters (i.e., additionally adjusted for ^a^OCT horizontal tilt angle and ovality index, ^b^optic disc torsion angle and ovality index, and ^c^optic disc torsion angle and OCT horizontal tilt angle).

*P* values in bold indicate statistical significance.
